# Glycine-Histidine-Lysine (GHK) Alleviates Astrocytes Injury of Intracerebral Hemorrhage via the Akt/miR-146a-3p/AQP4 Pathway

**DOI:** 10.3389/fnins.2020.576389

**Published:** 2020-10-28

**Authors:** Heyu Zhang, Yanzhe Wang, Ling Lian, Cheng Zhang, Zhiyi He

**Affiliations:** ^1^Department of Neurology, The First Affiliated Hospital, Sun Yat-sen University, Guangzhou, China; ^2^Guangdong Provincial Key Laboratory of Diagnosis and Treatment of Major Neurological Diseases, National Key Clinical Department and Key Discipline of Neurology, Guangzhou, China; ^3^Department of Neurology, First Hospital of China Medical University, Shenyang, China

**Keywords:** intracerebral hemorrhage, glycine-histidine-lysine, astrocytes, miR-146a-3p, AQP4

## Abstract

Intracerebral hemorrhage (ICH) is a major type of cerebrovascular disease with poor prognosis. Recent studies have shown that Glycyl-l-histidyl-l-lysine (GHK) is a kind of natural human tripeptide which could inhibit inflammation and against neurodegenerative diseases, but neither its role nor the mechanisms in ICH have yet been explicit. Currently, we investigated the possible strategies of GHK on ICH injury. Neurological deficit scores, brain water content, Nissl staining, and aquaporin 4 (AQP4) immunohistochemistry were detected in different groups of rats. The expression of microRNAs (miRNAs) was examined by real-time PCR. Inflammatory factors were detected using enzyme-linked immunosorbent assay (ELISA). Cell viability and cell proliferation were detected by Cell Counting Kit-8 (CCK-8). Matrix metalloproteinase 2 (MMP2), MMP9, tissue inhibitors of metalloproteinase-1 (TIMP1), AQP4 expression were detected/assessed using western blot. We observed that 5 and 10 μg/g of GHK improved neurological recovery by significantly reducing brain water content, improving neurological deficits, and promoting neuron survival. Besides, GHK alleviated inflammatory reaction and downregulated AQP4 expression. Furthermore, the effects of GHK on astrocyte were associated with the upregulation of miRNA-146a-3p, which partially regulated the expression of AQP4. Our results demonstrated that the phosphatidylinositol 3-kinase (PI3K)/AKT pathway participated in the GHK-induced upregulation of miR-146a-3p and miR-146a-3p/AQP4 interaction plays a role in the injury following ICH. These findings suggested that GHK could provide a novel therapeutic strategy for ICH.

## Introduction

Stroke is a leading cause of disability and mortality globally. Intracerebral hemorrhage (ICH) accounts for 15–20% of all stroke incidents, with the highest mortality in all subtypes of stroke, leading to serious social burden (Huang et al., 2019; Lo and Teitelbaum, 2020). In ICH, the initial injury by the rupture of vessels and the compression of the expanding hematoma is mostly followed by secondary damage, which involves hemoglobin cytotoxicity, microglial activation, and inflammatory cell infiltration in the perihematomal region (Yang et al., 2020). The concurrent effects of these events are issued in massive neuron apoptosis causing pejorative and sustained brain damage after ICH (Mohammed Thangameeran et al., 2020). However, unlike an ischemic stroke, there is no effective treatment for ICH. In this respect, an efficient and novel agent for preventing neuron death after ICH is necessary.

Neuron apoptosis occurs in ICH is the major reason for the poor prognosis following ICH injury (Zille et al., 2017). As a prominent cell type in the brain, astrocytes play an important role followed brain injury (Clarke and Barres, 2013). Several studies for protecting neurons by manipulating astrocytes have been revealed to be effective, and the role of astrocytes as both neuroprotective and exacerbating injury in ICH was reviewed recently (Barreto et al., 2011; Min et al., 2015). The reason astrocytes are used as therapeutic targets is that neurons cannot survive independently from astrocytes (Bylicky et al., 2018). Moreover, astrocyte viability is maintained stronger than neurons in the stroke. On this account, targeting surviving astrocytes offers an inestimable opportunity to recover the function of neurons and blood vessels in the process of ICH (Liu and Chopp, 2016). However, the explosive swelling of astrocyte contributes to its dysfunction and assistants with aquaporins (Thomas et al., 2004; Qiu et al., 2015). As a member of the aquaporins, aquaporin 4 (AQP4) is abundant in astrocytes processes and responsible for brain edema (Chu et al., 2016). Accumulating evidence has shown that inhibiting the expression of AQP4 could improve recovery of neurological function, against neuron apoptosis and inflammation response, while brain edema alleviated simultaneously (Tang and Yang, 2016). However, inhibitors for AQP4 have not been determined. Therefore, it is essential to explore an agent that could effectively regulate AQP4.

Glycyl-l-histidyl-l-lysine (GHK) is a kind of human tripeptide consist of GHK. It was first isolated by Pickart in 1973 that naturally occurring in plasma, saliva, and urine. Subsequent studies established that GHK and its copper (II)-chelated form (GHK-Cu) improved the process of regeneration, anti-inflammatory and anti-oxidant action (Pickart, 2008; Pickart et al., 2015). Furthermore, it has been convinced as a probable peptide for treating chronic obstructive pulmonary disease, attenuating skin inflammation, and inhibiting metastatic colon cancer (Gruchlik et al., 2012; Meiners and Eickelberg, 2012; Pickart et al., 2012). Also, studies have indicated that GHK enhances trophic factors secretion of mesenchymal stem cells, and exerts neuroprotective effects against neurodegenerative diseases by inhibiting inflammation, alleviating oxidative damage, even modulating the iron levels (Miller et al., 1990; Jose et al., 2014). Our previous study has found that GHK could alleviate neuron injury via VEGFA (Zhang H. et al., 2018). However, the effect and mechanism of GHK on ICH secondary injury which is related to glial cells is indecisive and should be further explored.

In this study, we studied the potential role of GHK in ICH rat models. We found that different concentrations of GHK could improve rats’ neurological function recovery, while alleviated brain edema, inflammation reaction, and down-regulated the level of AQP4 through miR-146a-3p after ICH. Furthermore, phosphatidylinositol 3-kinase (PI3K)/AKT pathway involves in the treatment of GHK in ICH. These results provide a novel therapeutic approach to ICH.

## Materials and Methods

### Animals

Adult male Wistar rats (between 250 and 280 g) were purchased from Changsheng Biotechnology Co., Ltd. Rats were carefully kept in an environmentally controlled room (22–25°C, 50% humidity) under a 12 h day-night rhythm. Food and water could be obtained *ad libitum*.

One hundred and twenty rats were randomly divided into five groups: (1) Sham group, 24 rats experienced the same surgical procedures as rats in the control group without collagenase VII injection. (2) Control group, 24 rats underwent the collagenase VII-induced ICH and injected vehicles intraperitoneally (i.p.) when the treatment groups were administered GHK. (3) GHK with 95% purity, was purchased from Chinapeptids Biotechnology Co., Ltd. (Zhou et al., 2017). GHK groups (GHK 1 μg/g, GHK 5 μg/g, and GHK 10 μg/g), rats in these groups were injected with GHK after the administration of collagenase VII. Twenty-four rats in each group were randomly divided into four groups by a researcher who was blind with the neurological deficits of these rats. Six rats were decapitated to obtain fresh brain tissue samples for water content. Six rats were perfused with fixative for histological preparation and analysis of the brains. Six rats were used for the neurological deficits scores until 7 days after ICH. Six rats were anesthetized by intraperitoneal injection of 2% pentobarbital intraperitoneally. Before they were decapitated, 300 mL physiological saline was administered to perform cardiac perfusion. A coronal cut was made with the pinhole as the center. The nasal side of perihematomal tissues in basal ganglia, which were prepared for biochemical analyses, were placed in liquid nitrogen for 30 min before storage at −80°C in enzyme-free tubes (Pei et al., 2016). All data were collected and analyzed by a researcher who was blind with the groups.

### Collagenase VII-Induced Intracerebral Hemorrhage Model

Intracerebral hemorrhage was induced via stereotactic administration of 0.25 U/μl bacterial collagenase type VII (Sigma, United States), as Rosenberg described (Rosenberg et al., 1990). Rats in the GHK treatment group were intraperitoneally injected with GHK every 24 h dissolved in saline for 3 days after the administration of ICH, while those in the control group were treated with equal volumes of vehicle.

### Brain Water Content

Rats were anesthetized, and the brains were collected 3 days following ICH. The wet weight of each sample was immediately measured by an electronic balance, following which the brains were dried at 100°C for 24 h to obtain the dry weight. Water content was calculated according to the following formula: [(wet weight – dry weight)/(wet weight) × 100 (%)] (Lee et al., 2008).

### Evaluation of Neurological Deficits

Neurological deficits were evaluated using a five-point scale established by Rosenberg et al. (1990), The extent of circling of rats was graded from 0 (no circling) to 4 (always circled) at 3 and 7 days after ICH induction.

### Immunohistochemistry and Nissl Staining

Immunohistochemistry experiments were performed using the UltraSensitiveTM SP (Mouse/Rabbit) immunohistochemistry Kit (Mixim, Fu Zhou, China), following the manufacturer’s instructions. The samples were fixed for 24 h by 4% paraformaldehyde and dehydrated using 70–100% alcohol. Paraffin-embedded brains were cut into 5 μm sections. The sections were suffered to antigen retrieval under high temperature and pressure conditions, following which they were treated with the endogenous peroxidase blockers and then blocked in serum for an hour. The sections were incubated in 1:200 AQP4 antibody overnight. Subsequently, the sections were incubated with secondary antibody (goat anti-rabbit), rinsed with phosphate buffer saline (PBS), stained with DAB, and hematoxylin and eosin (H&E) staining, dehydrated, transparentized, and mounted before microscopic analysis. We randomly selected 5 visual fields at the nasal side of perihematoma in basal ganglia, positive cells were discerned and analyzed by ImageJ software. For Nissl staining, the sections were subjected to cresyl violet acetate for 15 min at 23–25°C, rinsed in PBS, dehydrated, transparentized, and mounted before microscopic analysis.

### Immunofluorescence Staining

The samples were fixed with 4% paraformaldehyde for 24 h and dehydrated using a 10–30% saccharose solution. OCT embedded brains were cut into 10 μm sections, the sections were rinsed three times with PBS, blocked for 1 h in 8% bovine serum albumin (BSA) dissolved in 0.01 M phosphate buffer (PB) containing 0.5% Triton-X100. Then incubated at 4°C overnight with anti-glial fibrillary acidic protein (GFAP) (cell signaling technology, 80788) diluted 1:100 in 0.01 M PB containing 1% BSA and 0.1% Triton-X100. After rinsing with PBS, all sections were incubated at room temperature for 2 h with IgG goat anti-rabbit (Thermo Fisher Scientific, Alexa Fluor 488) diluted 1:200 in 0.01 M PB containing 1% BSA and 0.3% Triton-X100. Nuclei were stained with 200 nM 4,6-diamidino-2-phenyl-indole (DAPI) for 5 min. Sections were viewed via a fluorescence microscope.

### Cell Culture and *in vitro* Model of Hemorrhagic Toxicity

Astrocytes and 293T cells (Chinese Academy of Sciences, Shanghai) were cultured in dulbecco’s modified eagle medium (DMEM) (Gibco, UA) added 10% fetal bovine serum (Gibco, Australia), following which they were incubated at 100% humidity in an environment containing 5% CO_2_. To imitate hemorrhage toxicity *in vitro*, the model was constructed by applying 100 μM hemin to Astrocytes (Huang et al., 2002; Zille et al., 2017).

### Synthetic RNA Oligonucleotides and Transfection

We purchased miRNA-146a-3p mimics, miRNA-146a-3p inhibitors, and a non-sense sequence for use as a miRNA negative control (NC) from Genepharma (Shanghai, China) (Supplementary Table 2). Astrocytes were transfected with Lipofectamine 2000 (Invitrogen, United States), according to the manufacturer’s instructions.

### RNA Extraction and Quantitative Reverse Transcription-Polymerase Chain Reaction (qRT-PCR)

Total RNA was extracted from cellular and cerebral samples using TRIzol reagent (Invitrogen, Carlsbad, CA, United States). Reverse transcription of miRNA was performed using the Hairpin-it miRNAs qPCR Quantitation Kit (GenePharma, Shanghai, China), following the manufacturer’s protocol. T ABI 7500 system (Thermo Fisher Scientific, Carlsbad, CA, United States) was using for real-time PCR. The relative miRNA expression of each gene was normalized to the expression of U6 RNA. The primers were synthesized by Sangon Biotech (Shanghai). All primer sequences are listed in Supplementary Table 1.

### Western Blot Analysis

Cellular and cerebral samples were lysed in RIPA (Beyotime, China) containing inhibitor cocktail (Roche, Germany) for 20 min, following which the lysates were centrifuged (12,000 × g) at 4°C for 15 min. Protein concentration was determined using a BCA Protein Kit (Thermo, Rockford, IL, United States). Sample lysates were separated via 4–14% sodium dodecyl sulfate-polyacrylamide gel electrophoresis (SDS-PAGE), following which the proteins were transferred to polyvinylidene difluoride (PVDF) membranes. The membranes were then incubated in 1% bovine serum albumin (BSA) solution with the following primary antibodies overnight at 4°C: anti-AQP4 (Abcam, ab18956), anti-MMP2 (Abcam, ab37150), anti-MMP9 (Abcam, ab38898), anti-TIMP1 (Abcam, ab109125) and anti-beta actin (Abcam, ab8227). The membranes were then incubated with secondary antibody for 1 h at room temperature. We analyzed the relative signal densities by ImageJ software, and β-actin density was used as the internal control.

### Cell Counting Kit-8 (CCK-8) Assay for Cell Viability and Cell Proliferation

Cell viability was determined using the CCK-8 solution (Beyotime, China), under the manufacturer’s instructions. Approximately 5 × 10^3^ cells were seeded in each well of a 96-well plate overnight, following which the cells were applied hemin for 18 h, GHK was added to the culture medium simultaneously. Cell proliferation detection was according to the manufacturer’s instruction. In brief, 2 × 10^3^ cells were seeded in each well of a 96-well plate for 4 h, then GHK was added and this time is known as 0 h, and the cells were cultured for 24 and 48 h for detecting cell proliferation. A total of 10 μl of CCK-8 solution was added to each well, and the cells were incubated for 1 h in the incubator. The absorbance of each well was quantified at 450 nm using an automated ELISA reader (SpectraMax^®^ M5, Molecular Devices, United States). Cell viability was calculated as follows: (A450 of experimental wells/A450 of normal wells) × 100%.

### Lactate Dehydrogenase (LDH) Assay for Cytotoxicity

Cytotoxicity was determined using an LDH Assay Kit (Beyotime, China), in accordance with the manufacturer’s instructions. Following applying hemin for 18 h, the supernatant was collected and transferred to 96-well plates to detect LDH release. The absorbance of each sample was measured at 490 nm using an automated ELISA reader (SpectraMax^®^ M5, Molecular Devices, United States). Percent cytotoxicity was calculated as follows: (A490 of experimental culture medium-A490 of normal medium)/(A490 of maximum LDH release-A490 of normal wells) × 100%.

### Measurement of the TNF-α and IL-1β by ELISA

The TNF-α and IL-1β levels in the perihematomal were determined using ELISA kits (USCN Life Science Inc., Wuhan, China) according to the manufacturer’s instructions. The absorbance of each sample was quantified at 450 nm using an automated ELISA reader (SpectraMax^®^ M5, Molecular Devices, United States).

### Dual-Luciferase Assays

The pmirGLO Dual-Luciferase miRNA target expression vectors were constructed by Genepharma (Shanghai, China). 1 × 10^4^ 293T cells were seeded in each well of a 24-well plate overnight, following which they were co-transfected with wild-type and mutant AQP4 promoter-luciferase plasmids (0.1 μg per well) and 0.4μg miR-146a-3p mimics or NCs using Lipofectamine 2000 (Invitrogen, United States). The pmirGLO-wt-AQP4 and pmirGLO-mt-AQP4 were purchased from Genepharma Transfection efficiency was standardized based on that of TK activity. Luciferase activity was quantified using a dual-luciferase assay system (Promega, E1910).

### Statistical Analysis

Each assay was repeated independently at least three times to ensure the reliability of this study and the measurement data are expressed as the mean ± SD. We also performed a prior power analysis using the G^∗^Power 3.1.9.2 software with a significance level of 5% and a power level > 0.9 to ensure we used an adequate number of animals per group. All data were analyzed using SPSS 23.0 software (IBM Corp., Armonk) and Graphpad Prism 5.0 (GraphPad Software). Comparisons among multiple groups were performed using one-way ANOVA followed by a Bonferroni or SNK test. Behavioral data were analyzed using the Kruskal–Wallis test with Dunn’s test for multiple comparisons. Coefficients of correlation were analyzed by the Pearson correlation method. The demarcation of statistical significance was set at *p* < 0.05.

## Results

### GHK Alleviated Neurological Deficits and Promoted Neuron Survival Following ICH

**FIGURE 1:**
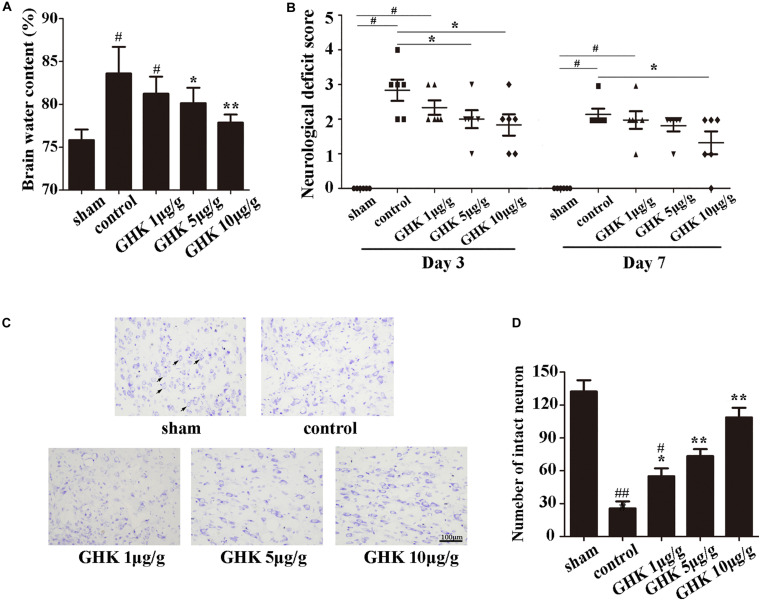
GHK alleviated neurological injury and neuronal apoptosis following intracerebral hemorrhage (ICH). **(A)** GHK delivery via the intraperitoneal route decreased water content in the brain 72 h after ICH. **(B)** Analyses of neurological deficits revealed that treatment with both 5 μg/g and 10 μg/g GHK facilitated neurological recovery at 3 and 7 days after ICH. **(C)** Nissl staining *in vivo*. Paraffin slices (5 μm) from the control and GHK groups were stained with cresyl violet acetate. (arrows are pointing the intact neurons). **(D)** The number of the intact neuron. (*n* = 6. ^#^*p* < 0.05 vs. sham; *p < 0.05 vs. control; **p < 0.01 vs. control, ^##^*p* < 0.01 vs. sham).

To investigate the potential role of GHK post-ICH, we isolated the rat brain 3 days after ICH. We measured brain water content and intact neuron number in different groups of rats. Neurological deficits were detected 3 and 7 days after ICH. Our results indicated that GHK significantly decreased brain water content (Figure 1A) and relieved the neurological deficits in ICH rats (Figure 1B). Numbers of survival neurons were significantly increased in rats treated with GHK, relative to findings observed in control rats (Figures 1C,D).

### GHK Ameliorated Inflammation and Metalloprotease/Anti-metalloprotease Imbalance in ICH

**FIGURE 2:**
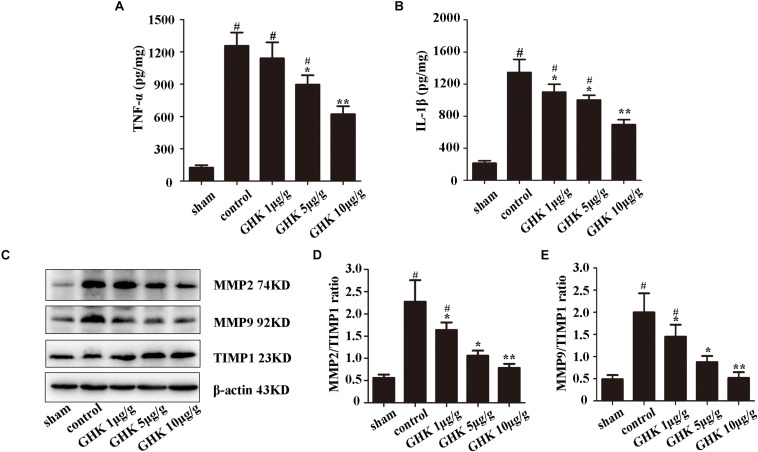
GHK alleviated inflammation and metalloprotease/anti-metalloprotease imbalance in ICH. **(A)** The expression level of TNF-α. **(B)** The expression level of IL-1β. **(C–E)** The expression of MMP2, MMP9, and TIMP1 in different groups of rats. (*n* = 6. ^#^*p* < 0.05 vs. sham; *p < 0.05 vs. control; **p < 0.01 vs. control).

To investigate the effect of GHK following ICH, we estimated the level of TNF-α and IL-1β in ICH brain samples using ELISA (Figures 2A,B). The expression of MMP2, MMP2, TIMP1 was detected using western blot (Figures 2C–E). The result has shown that GHK significantly decreased the expression of TNF-α and IL-1β, which are the crucial factors in the inflammation of ICH. Furthermore, the expression of MMP2 and MMP9 downregulated while the expression of TIMP1 increased. These findings demonstrated that GHK could alleviate the inflammation and reverse the imbalance of metalloprotease/anti-metalloprotease following ICH. However, the activation of metalloproteases is related to astrocytes injury in ICH.

### GHK Alleviated Astrocytes Injury Following ICH and Down-Regulated AQP4 Expression

**FIGURE 3:**
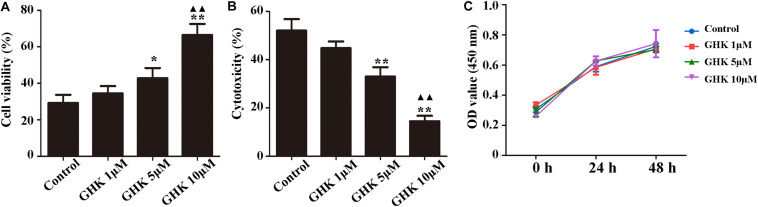
GHK improved the survival of astrocytes. **(A)** GHK increased the viability of astrocytes in dose-dependent manner. **(B)** GHK decreased the cytotoxicity of astrocyte. **(C)** GHK not significantly promoted astrocytes proliferation. (One-way ANOVA was used for repeated measurements. Data are shown as the mean ± SD, *n* = 3. *p < 0.05 vs. control; **p < 0.01 vs. control; ^▲▲^*p* < 0.05 vs. GHK 5 μM).

**FIGURE 4:**
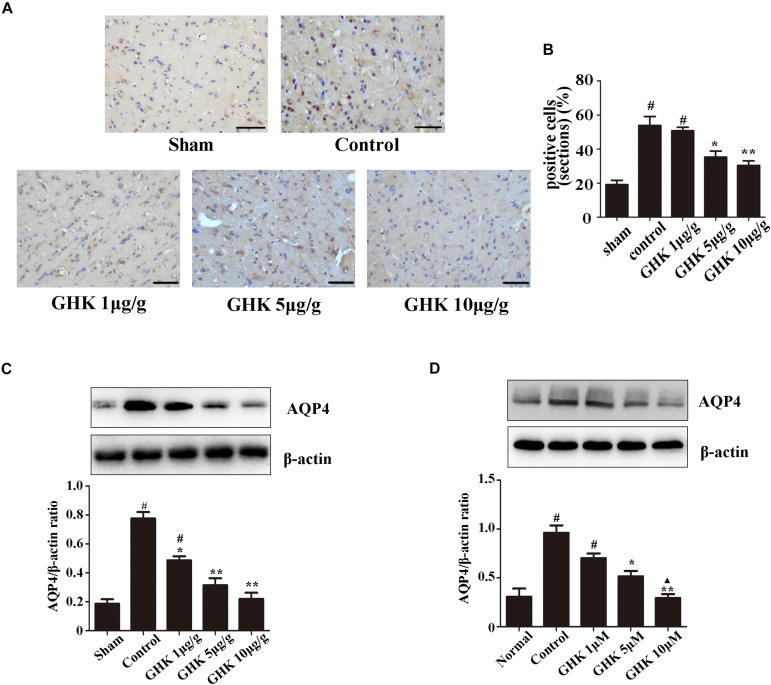
GHK down-regulated the expression of AQP4. **(A)** Immunohistochemistry analysis of AQP4 expression. Paraffin slices (5 μm) from the control and GHK groups were stained with AQP4. **(B)** Quantification of AQP4 positive cells (*n* = 6. ^#^*p* < 0.05 vs. sham; *p < 0.05 vs. control; **p < 0.01 vs. control). **(C)** Western blot analysis illustrating the effect of GHK on AQP4 expression in rats perihematomal tissue (*n* = 6. ^#^*p* < 0.05 vs. sham; *p < 0.05 vs. control; **p < 0.01 vs. control). **(D)** The expression of AQP4 in astrocytes under hemin-stimulation. (One-way ANOVA was used for repeated measurements. Data are shown as the mean ± SD, *n* = 3. *p < 0.05 vs. control; **p < 0.01 vs. control; ^▲^*p* < 0.05 vs. GHK 5μM).

Astrocytes are the major glial cells in the brain and charge of brain homeostasis maintenance. To investigate the effect of GHK on astrocytes following ICH, we evaluated the cell viability and cell proliferation *in vitro* by CCK-8 (Figures 3A,C), cytotoxicity by Lactate dehydrogenase (LDH) (Figure 3B). We also detected the activation of astrocytes around the hematoma, the expression of GFAP without significant difference (Supplementary Figure 2). The results showed that GHK significantly increased the survival of astrocytes, but no significant difference in proliferation. Moreover, we detected the expression of AQP4 using immunohistochemistry (Figures 4A,B) and western blot (Figures 4C,D). The results determined the level of AQP4 decreased following the increase of GHK concentration.

### AQP4 Was a Functional Target Gene of miRNA-146a-3p

**FIGURE 5:**
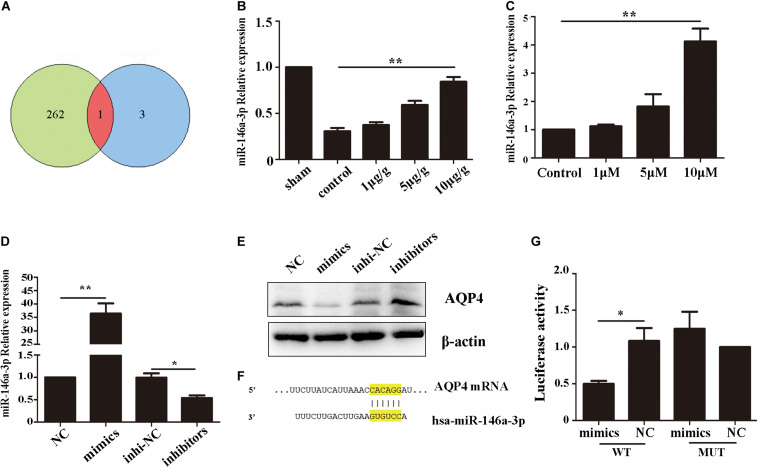
AQP4 was a functional target gene of miR-146a-3p. **(A)** The intersection of potential miRNAs of AQP4 and miRNAs downregulated in ICH patients. **(B)** Level of miR-146a-3p in different concentration GHK-treated ICH rat perihematomal tissue, relative to control values (*n* = 6, **p < 0.01 vs. control). **(C)** RT-qPCR assays for miR-146a-3p in astrocytes under hemin-stimulation as compared with control group (*n* = 3, **p < 0.01). **(D)** The expression level of miR-146a-3p after mimics, inhibitor and NCs transfected (*n* = 3, *p < 0.05, **p < 0.01). **(E)** The expression of AQP4 following miR-146a-3p mimics, inhibitor and NCs transfected. **(F)** Binding sites for miR-146a-3p and AQP4. **(G)** Levels of luciferase activity in 293T cells co-transfected with miR-146a-3p and AQP4. *p < 0.05.

To investigate the reason of AQP4 decreased after GHK treatment, we predict the potential target microRNAs (miRNAs) of AQP4 (Supplementary Table 1) and got intersection elements with miRNAs down-regulated in ICH patients (Zhu et al., 2015; Figure 5A). Then we detected the expression of miR-146-3p in astrocytes using real-time PCR. The result demonstrated that miR-146a-3p significantly upregulated followed GHK treatment (Figures 5B,C). To confirm the prediction, we found the binding region of miR-146a-3p and AQP4 (Figure 5F) and transfected miR-146a-3p into astrocytes. Before the experiment, the expression level was detected by real-time PCR (Figure 5D). We found that miR-146a-3p expression revealed a completely contrary trend to AQP4 expression (Figure 5E). Furthermore, dual-luciferase assays were employed to illuminate the interaction of miR-146a-3p with AQP4 mRNA. A significant decrease in the relative luciferase activity was observed in the miR-146a-3p + pGLO-wt-AQP4 group compared to the other groups (*p* < 0.05) (Figure 5G).

GHK Increases miR-146a-3p Levels Through the PI3K/Akt Pathway and miR-146a-3p Inhibitor Reverses GHK-induced AQP4 Reduction in Astrocytes.

**FIGURE 6:**
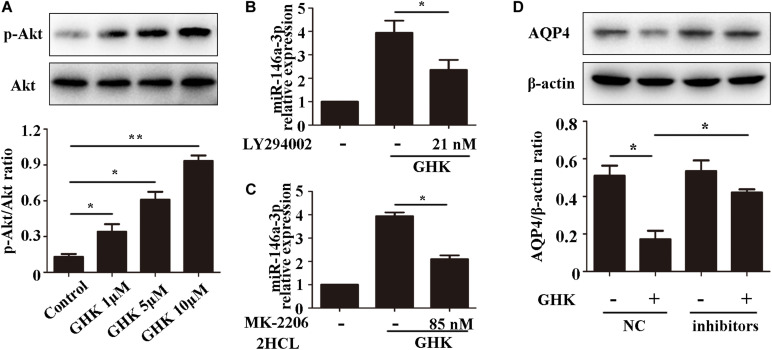
GHK increases miR-146a-3p levels through the PI3K/Akt pathway and miR-146a-3p inhibition reverses AQP4 decrease induced by GHK in astrocytes. **(A)** Expression of p-Akt and Akt in astrocytes with different concentration of GHK (*n* = 3, *p < 0.05, **p < 0.01). **(B,C)** Expression of miR-146a-3p in astrocytes with PI3K inhibitor LY294002 and Akt inhibitor MK-2206 2HCL were incubated with 10 μM GHK simultaneously (*n* = 3. *p < 0.05 vs. GHK. Analysis using ImageJ). **(D)** Protein levels of AQP4 in astrocytes treated with GHK and the miR-146a-3p NC or inhibitor. (*n* = 3, *p < 0.05. Analysis using ImageJ).

**FIGURE 7:**
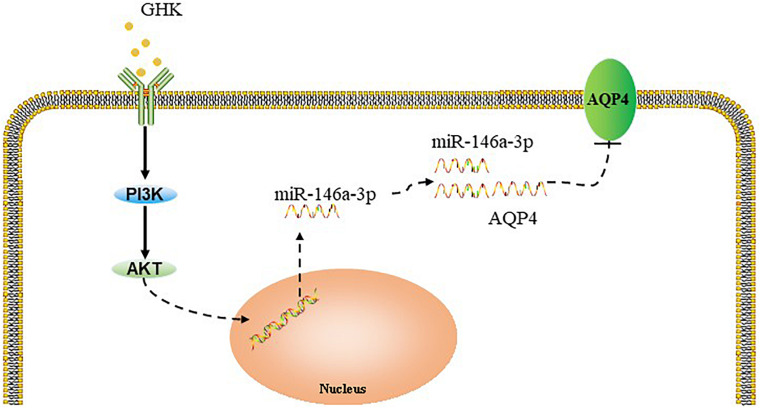
Summary model of the down-regulation of AQP4 induced by GHK via Akt/miR-146a-3p/AQP4 in astrocytes.

Previous studies have demonstrated that the PI3K/Akt pathway plays important role in ***ICH*** injury. Cui et al. (2015, 2017) has revealed that the activation of the PI3K/Akt pathway acted a protective effect in ICH. Also, some researchers have demonstrated inhibiting PI3Kleaded to a dose-dependent downregulation in the expression of mature miR-146a (Jiang, 2014). To study the pathway of which GHK increase miR-146a-3p levels, different concentrations of PI3K/Akt inhibitor added to the medium of astrocytes, which were stimulated with 10 μM GHK simultaneously (Figure 6A). We revealed that various concentrations of the PI3K inhibitor LY294002 and Akt inhibitor MK-2206 2HCL suppressed the up-regulation of miR-146a-3p levels induced by GHK in a concentration-dependent manner (Figures 6B,C). Furthermore, miR-146a-3p inhibitors can reverse the downregulation of AQP4 (Figure 6D). These results indicated that GHK decreases AQP4 expression via miR-146a-3p through the PI3K/Akt pathway (Figure 7).

## Discussion

GHK, a human tripeptide, natural and non-toxic, exhibits extraordinary effects with anti-inflammatory and neuroprotective effects in acute lung injury (Pickart et al., 2014; Park et al., 2016) and neurodegenerative diseases (Pickart et al., 2012, 2017). In our study, GHK protected against ICH injury by reducing brain edema and promoting neuron survival. Surprisingly, we found that intraperitoneally injected at a dose of 10 μg/g significantly alleviated neurological deficits until 7 days post-ICH, which suggested a dose of 10 μg/g can also play a role in the subacute phase post-ICH. These findings provided further evidence that GHK could be an available agent for ICH therapy. Our results demonstrated novel insights into the potential roles of GHK ICH secondary injury.

Intracerebral hemorrhage injury mainly results from the secondary injury followed hematoma formation including hemoglobin cytotoxicity and explosive inflammatory response, which mainly resulted in brain edema and neuron death (Shi et al., 2012; Zhang et al., 2014). Therefore, timely control of cytotoxic edema may contribute to alleviating hemorrhagic injury. We observed that GHK significantly alleviated the brain edema, neurological deficits, improved neuron survival and reduced the level of TNF-α and IL-1β. Furthermore, we found that GHK relieved the imbalance of matrix metalloproteinase (MMPs) synchronously. According to the previous study, the matrix metalloproteinase level was clearly evaluated after ICH, and the activation of MMP9 in astrocytes exacerbated ICH (Lattanzi et al., 2020). We believe that GHK may be an efficient agent for ICH by targeting astrocytes.

Accumulating evidence revealed that astrocytes play various homeostatic roles in the brain (Sofroniew and Vinters, 2010; Sticozzi et al., 2013). The activity of astrocytes is an important basis for the survival of neurons in brain injury (Ouyang et al., 2014). In addition to building the blood-brain barrier and secreting neural trophic factors, astrocytes are also responsible for brain edema and inflammation response after ICH (Chen-Roetling et al., 2015). In this study, we found that GHK could increase the viability and alleviate the cytotoxicity of astrocytes. These results showed that GHK significantly alleviated the injury of astrocytes due to ICH. Aquaporins play important roles in neural injury (Wang et al., 2015). AQP4 is one of the key transport channels in the central nervous system, which is abundant in astrocytes foot processes (Bonomini and Rezzani, 2010). Previous studies have demonstrated that AQP4 reactivity increase following ICH which plays a critical role in brain edema and cytotoxic cell death (Appelboom et al., 2015; Qiu et al., 2015). Increasing evidence indicates that inhibition of AQP4 alleviates the brain injury and improves the recovery of ICH (Zhong et al., 2013; Wang et al., 2015). We observed that the expression of AQP4 significantly declined with GHK treatment. However, the mechanism of GHK regulating AQP4 should be further explored.

Several studies of stroke have demonstrated that signal pathways regulate AQP4 expressions, such as the MAPK pathway and NF-κB pathway (Qi et al., 2011; Boldin and Baltimore, 2012). Moreover, several miRNAs, such as miR-29b and miR-130a, regulate AQP4 expression in ischemic stroke. miRNAs serve as an important constituent of non-coding RNAs. They bind to complementary sequences on target mRNAs, resulting in negative regulation through degraded target mRNA or repressed the translation (Beermann et al., 2016). Our previous results demonstrated that miR-146a-3p significantly decreased in ICH patients’ peripheral blood (Zhu et al., 2015). Ji et al. (2013) demonstrated that miR-146a regulated the level of SOD in H_2_O_2_ stimulated PC 12 cells. In addition, miR-146a-3p has been widely known as an important molecule in the astrocyte-related inflammatory response in neuropathy such as Alzheimer’s Disease and epilepsy (Aronica et al., 2010; Cui et al., 2010; Iyer et al., 2012). We found that AQP4 was a functional target gene of miR-146a-3p, and the level of miR-146a-3p was completely contrary to AQP4 following GHK treatment. Furthermore, the dual-luciferase activity of miR-146a-3p and AQP4 was significantly down-regulated, which indicated the direct effect of miR-146a-3p with AQP4. These results suggested that miR-146a-3p plays a role in GHK regulation of AQP4.

Accumulating evidence has indicated that the activation of the PI3K/Akt pathway involves the improvement of neurological functional recovery on ICH (Niu and Hu, 2017; Zhang W. et al., 2018). Jiang (2014) showed that PI3K/Akt pathway activation promoted the expression of miR-146a-3p in human alveolar epithelial cells, Walsh et al. (2015) reported that PI3K/Akt pathway involves in the regulation of miR-146a in T-cells. Moreover, existing evidence demonstrated that inhibiting the phosphorylation of PI3K could increase AQP4 expression, which suggested that PI3K/Akt involves in the regulation of AQP4 (Song et al., 2018). In this study, we observed that GHK increased the phosphorylation of Akt in astrocytes. Moreover, GHK upregulated the level of miR-146a-3p and decreased the expression of AQP4, while the LY294002 and MK-2206 2HCL could reverse these effects. Therefore, we assumed that the PI3K/Akt pathway was involved in the GHK-induced upregulation of miR-146a-3p and that the miR-146a-3p/AQP4 axis played a role in manipulating astrocytes following ICH injury.

Some limitations in our study must be documented. We used collagenase VII induced ICH model in this study which cannot completely simulate the pathophysiological process of ICH. We found that GHK could increase the phosphorylation of PI3K/Akt pathway, but we are not focus on the directly binding component. However, we focus on the effect of GHK on astrocytes only, rather than the interaction with neurons. Therefore, the specific role of GHK on ICH should be further explored.

## Conclusion

In conclusion, our findings demonstrated that GHK reduced brain edema, improved neurological recovery, and alleviated the inflammation in a rat model of ICH. In addition, we observed that GHK treatment downregulated AQP4 expression and decreased injury of astrocytes, likely via the upregulation of miR-146a-3p. Moreover, inhibition of the PI3K/Akt activation restrained the GHK-induced upregulation of miR-146a-3p and the downregulation of AQP4. Our findings suggested that GHK may become a novel therapeutic for ICH.

## Data Availability Statement

All datasets generated for this study are included in the article/Supplementary Material.

## Ethics Statement

All procedures were conducted in accordance with the regulations of the animal protection laws of China and approved by the Animal Ethics Committee of China Medical University (2012-38-1 and 2017008).

## Author Contributions

HZ: data curation, investigation, methodology, writing original draft, investigation. YW: data curation formal analysis, methodology, conceptualization. LL: visualization. CZ: writing—review and editing. ZH: funding acquisition, conceptualization, supervision, writing—review and editing. All authors contributed to the article and approved the submitted version.

## Conflict of Interest

The authors declare that the research was conducted in the absence of any commercial or financial relationships that could be construed as a potential conflict of interest.

## Supplementary Material

Supplementary Figure 1 The level of phosphorylate-Akt in brain tissue detected by western blotting.

Supplementary Figure 2 The expression of GFAP around the hematoma.

Supplementary Figure 3 Inhibition of AQP4 in astrocytes improve the survival of neurons.

Supplementary Figure 4 The expression of AQP4 in astrocytes treated with GHK and miR-146a-3p NC or mimics.

Supplementary Table 1 The target miRNAs of AQP4.

Supplementary Table 2 The Sequences of RNA oligos.

Supplementary Table 3 The sequences of primers.
