# Supplementary figures and images for: Glycine-Histidine-Lysine (GHK) Alleviates Astrocytes Injury of Intracerebral Hemorrhage via the Akt/miR-146a-3p/AQP4 Pathway
Source: Front Neurosci. 2020 Oct 28;14:576389. doi: 10.3389/fnins.2020.576389 (PMC7658812; doi:10.3389/fnins.2020.576389)

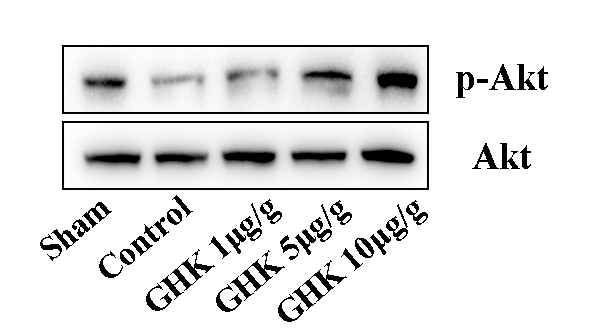

Supplement: Supplementary Figure 1 — The level of phosphorylate-Akt in brain tissue detected by western blotting. [file Image_1.TIF]

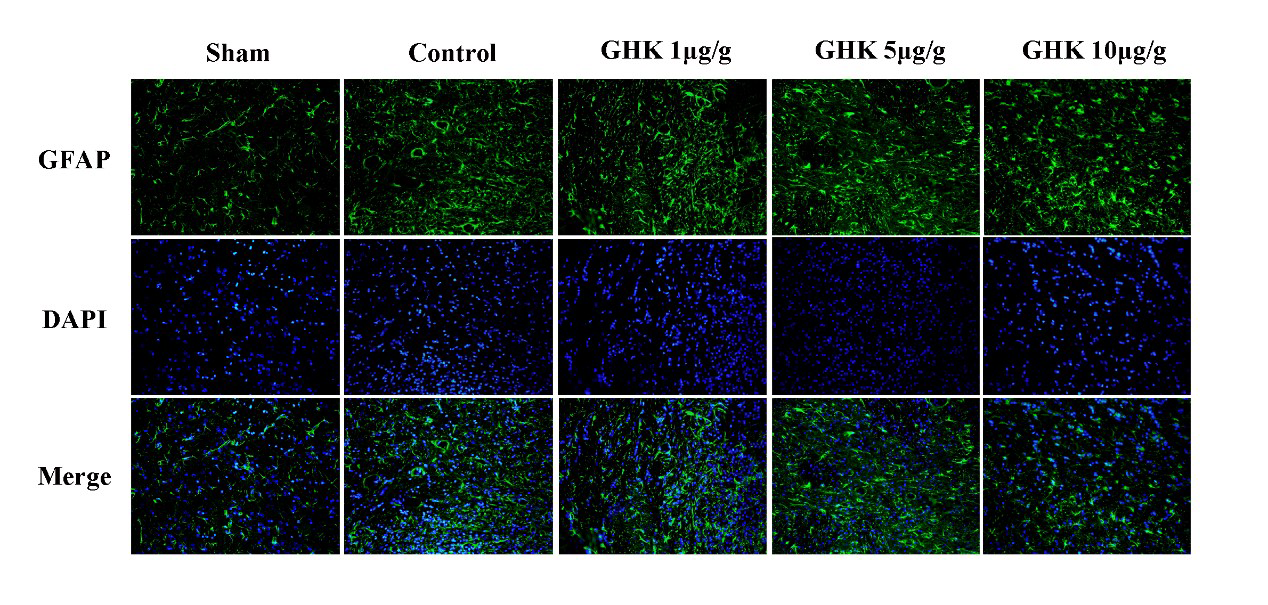

Supplement: Supplementary Figure 2 — The expression of GFAP around the hematoma. [file Image_2.TIF]

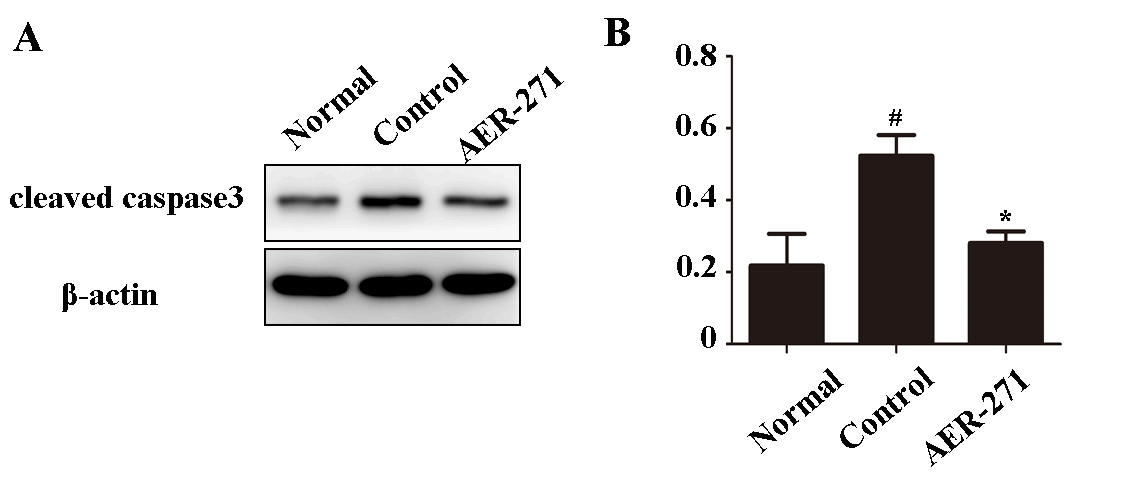

Supplement: Supplementary Figure 3 — Inhibition of AQP4 in astrocytes improve the survival of neurons. [file Image_3.TIF]

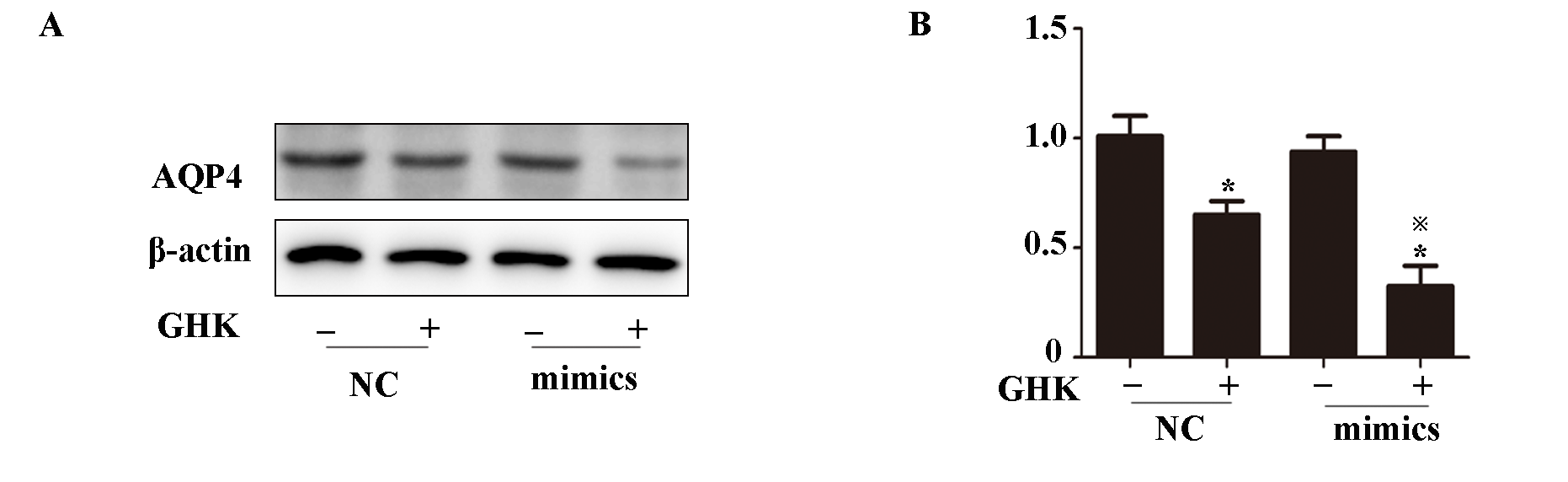

Supplement: Supplementary Figure 4 — The expression of AQP4 in astrocytes treated with GHK and miR-146a-3p NC or mimics. [file Image_4.TIF]
