# Supplementary material for: Glycine-Histidine-Lysine (GHK) Alleviates Astrocytes Injury of Intracerebral Hemorrhage via the Akt/miR-146a-3p/AQP4 Pathway
Source: Front Neurosci. 2020 Oct 28;14:576389. doi: 10.3389/fnins.2020.576389 (PMC7658812; doi:10.3389/fnins.2020.576389)
Supplement: Supplementary Table 2 — The Sequences of RNA oligos. [file Table_2.DOCX]

|  | Sequence |
| --- | --- |
| miRNA-146a-3p mimic | 5’-ACCUGUGAAGUUCAGUUCUUU-3’  5’-AAAGAACUGAACUUCACAGGU-3’ |
| miRNA-146a-3p inhibitor | 5’-AAAGAACUGAACUUCACAGGU-3’ |
| Negative control | 5’-UUCUCCGAACGUGUCACGUTT-3’  5’-ACGUGACACGUUCGGAGAATT-3’ |
| Inhibitor negative control | 5’-CAGUACUUUUGUGUAGUACAA-3’ |

**Table S2. The sequences of RNA oligoes**
