# Supplementary material for: Glycine-Histidine-Lysine (GHK) Alleviates Astrocytes Injury of Intracerebral Hemorrhage via the Akt/miR-146a-3p/AQP4 Pathway
Source: Front Neurosci. 2020 Oct 28;14:576389. doi: 10.3389/fnins.2020.576389 (PMC7658812; doi:10.3389/fnins.2020.576389)
Supplement: Supplementary Table 3 — The sequences of primers. [file Table_3.DOCX]

**Table S3. The sequences of primers**

|  | Forward | Reverse |
| --- | --- | --- |
| miR-146a-3p | 5’- GCGCGACCTGTGAAGTTCAG-3’ | 5’- AGTGCAGGGTCCGAGGTATT-3’ |
| U6 | 5′-ATTGGAACGATACAGAGAAGATT-3′ | 5′-GGAACGCTTCACGAATTTG-3′ |
